# TWEAK and LTβ Signaling during Chronic Liver Disease

**DOI:** 10.3389/fimmu.2014.00039

**Published:** 2014-02-13

**Authors:** Benjamin J. Dwyer, John K. Olynyk, Grant A. Ramm, Janina E. E. Tirnitz-Parker

**Affiliations:** ^1^School of Biomedical Sciences, CHIRI Biosciences Research Precinct, Curtin University, Bentley, WA, Australia; ^2^School of Medicine and Pharmacology, University of Western Australia, Fremantle, WA, Australia; ^3^Department of Gastroenterology, Fremantle Hospital, Fremantle, WA, Australia; ^4^Institute for Immunology and Infectious Diseases, Murdoch University, Murdoch, WA, Australia; ^5^Faculty of Medicine and Biomedical Sciences, The University of Queensland, Brisbane, QLD, Australia; ^6^QIMR Berghofer Medical Research Institute, Brisbane, QLD, Australia

**Keywords:** liver progenitor cells, hepatic stellate cells, regeneration, fibrosis, cancer, TWEAK, LTβ, NFκB

## Abstract

Chronic liver diseases (CLD) such as hepatitis B and C virus infection, alcoholic liver disease, and non-alcoholic steatohepatitis are associated with hepatocellular necrosis, continual inflammation, and hepatic fibrosis. The induced microenvironment triggers the activation of liver-resident progenitor cells (LPCs) while hepatocyte replication is inhibited. In the early injury stages, LPCs regenerate the liver by proliferation, migration to sites of injury, and differentiation into functional biliary epithelial cells or hepatocytes. However, when this process becomes dysregulated, wound healing can progress to pathological fibrosis, cirrhosis, and eventually hepatocellular carcinoma. The other key mediators in the pathogenesis of progressive CLD are fibrosis-driving, activated hepatic stellate cells (HSCs) that usually proliferate in very close spatial association with LPCs. Recent studies from our group and others have suggested the potential for cytokine and chemokine cross-talk between LPCs and HSCs, which is mainly driven by the tumor necrosis factor (TNF) family members, TNF-like weak inducer of apoptosis (TWEAK) and lymphotoxin-β, potentially dictating the pathological outcomes of chronic liver injury.

## Introduction

The liver possesses an extraordinary ability to orchestrate hepatocyte-mediated regeneration from acute injuries such as tissue resection or hepatic necrosis. However, hepatocyte proliferation is impaired or ablated by severe and chronic injury, dictating the need for an alternative liver regeneration pathway. Through a complex network of chemical and cellular mediators, the liver regenerates via the activation of a progenitor cell compartment, which retains proliferative and restorative capacity under severe injury conditions. Early chronic liver disease is typified by hepatocellular necrosis, hepatic inflammation, and release of immunomodulatory molecules by resident and recruited inflammatory cells, and dying hepatocytes, which activate the regenerative and fibrogenic wound healing responses.

The regenerative response consists of the “ductular reaction” (DR), in which bile ductules and liver progenitor cells (LPCs) proliferate from the Canals of Hering, the interface between the hepatocyte canaliculi and the biliary tree (1), resulting in biliary hyperplasia and the appearance of intermediate hepatocytes (2). Recent lineage-labeling studies have demonstrated that LPCs arise from a population of Sox9-expressing ductal cells that are activated to proliferate and differentiate into hepatocytes under certain chronic liver injury conditions (3, 4). This expansion is initiated and maintained by inflammatory cell-derived stimuli such as tumor necrosis factor (TNF) (5, 6) and interferon (IFN)-γ (7). Importantly, IFNγ has been shown to promote LPC expansion and inhibit proliferation of hepatocytes in concert with TNF stimulation (7). Additionally, transforming growth factor (TGF)-β suppresses liver epithelial cell proliferation. However, LPCs are significantly less sensitive to TGFβ-mediated growth inhibition during chronic liver injury and *in vitro* (8). In addition to stimuli from inflammatory cells, dying hepatocytes release hedgehog ligand (9), which has recently been shown to signal via the canonical Smoothened-dependent signaling cascade in primary cilium-positive LPCs, promoting their proliferation (10). Thus, the consequence of chronic hepatocyte injury and the subsequent inflammatory response is a liver microenvironment, which supports LPC expansion, while disabling hepatocyte-mediated regeneration.

LPC expansion occurs almost synchronously with fibrogenic wound healing, which is primarily driven by the action of hepatic stellate cells (HSCs). During chronic liver disease, quiescent HSCs are “activated” by inflammatory cytokines and begin to express α-smooth muscle actin (αSMA), signifying their transition to a myofibroblastic phenotype (11). Following this transition, activated HSCs drive fibrosis by depositing extracellular matrix (ECM) proteins, which assists to control LPC proliferation and differentiation. Accumulation of ECM is supported by the expression of tissue inhibitor of metalloproteinase (TIMP) proteins that inhibit matrix metalloproteases (MMPs), which function to degrade ECM proteins (12). Activated HSCs further reinforce regenerative and repair responses by expressing chemotactic factors such as intercellular adhesion molecule 1 (ICAM-1) and regulated upon activation, normal T-cell expressed, and secreted (RANTES), which attract additional inflammatory and progenitor cells to the site of injury (13). If the hepatic insult is resolved, LPCs mature to replace the lost epithelial cell types, hepatocytes, and/or cholangiocytes, depending on the underlying pathology. At the same time fibrosis recedes restoring structural and functional integrity of the liver. However, if injury persists, the regenerative and wound healing processes spiral out of control and become pathological. Chronic stimulation of HSCs results in fibrosis from excessive matrix deposition. With further impaired regeneration, this may progress to cirrhosis and liver failure. Furthermore, the prolonged stimulation of LPCs by pro-proliferative/survival cytokines from inflammatory cells generates a niche favoring the accumulation of genetic and epigenetic alterations, which can lead to the malignant transformation of LPCs and ultimately the formation of hepatocellular carcinoma (HCC).

A combination of association studies in patients and pathway manipulation experiments in rodents implicate LPCs as key regulatory cells in progressive chronic liver injury. LPCs are observed in many diseases with a predisposition to HCC, including chronic hepatitis B (HBV) and C virus (HCV) infection (14, 15), non-alcoholic fatty liver disease (16), alcoholic liver disease, and genetic hemochromatosis (14). Importantly, the numbers of LPCs increase as liver fibrosis progresses to cirrhosis, regardless of the underlying liver pathology (14, 17). Since LPCs proliferate at various stages of human liver tumorigenesis, ranging from preneoplastic lesions (18) to well-developed HCCs (19–21), they have been suggested as causative players during tumor development and maintenance. Supporting evidence comes from murine intervention studies where selective inhibition of c-kit^+^ LPCs by imatinib mesylate resulted in reduced tumor formation (6). These studies suggest that LPCs either represent direct cellular precursors of HCC or they crucially influence disease development by regulating other contributing cells such as fibrosis-driving HSCs. Either way they represent ideal chemotherapeutic targets for HCC prevention strategies in chronic liver injury.

**Figure 1:**
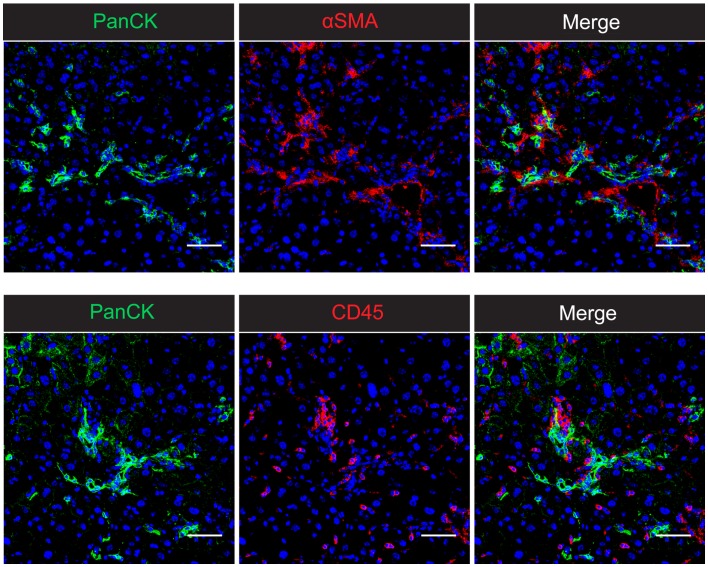
**The intrahepatic wound healing and regenerative niche in CDE-induced chronic liver injury in mice**. Confocal microscopy of 2-week CDE-injured mouse liver shows the close association of PanCK^+^ liver progenitor cells with both αSMA^+^ activated hepatic stellate cells and CD45^+^ inflammatory cells. The spatial proximity of all three cell types suggests the potential for cellular cross-talk and co-regulation of the inflammatory, fibrogenic, and progenitor cell responses. Nuclei are stained with DAPI (blue). Scale bars represent 50 μm.

**Figure 2:**
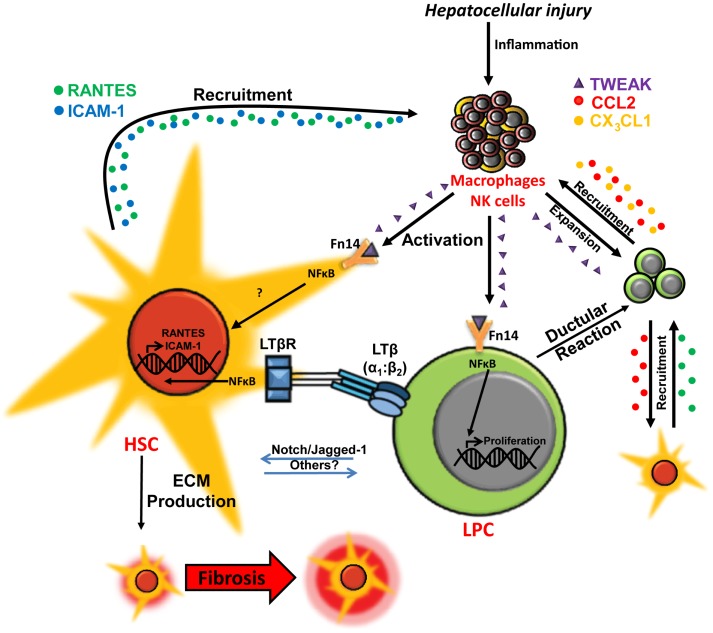
**Co-regulation of hepatic wound healing and regenerative responses via TWEAK- and LTβ-mediated cellular cross-talk**. In chronic liver injury liver progenitor cell (LPC) proliferation is induced via macrophage/natural killer (NK) cell-produced TWEAK activation of the NFκB pathway. Interaction between LPCs and activated hepatic stellate cells (HSCs)/myofibroblasts via Notch pathway and other potential mediators may drive LPC differentiation to cholangiocytes. LPCs and ductular reaction cells produce chemokines, such as CCL2 (red) and CX_3_CL1 (orange), which promote chemotaxis of inflammatory/myofibroblastic cells. Interactions between lymphotoxin-β (LTβ) expressed on LPCs and the LTβ receptor (LTβR) on activated HSCs triggers NFκB-driven expression of chemotaxis-associated factors ICAM-1 (blue) and RANTES (green) by HSCs, which may play a role in mediating recruitment of LPCs, HSCs, and leukocytes for wound healing, fibrogenesis and regeneration during chronic liver injury (13, 22–27).

The processes of inflammation, fibrosis, and LPC induction are tightly regulated and occur in close spatial association (Figure 1), suggesting the potential for cellular communication (Figure 2). Cross-talk between the hepatic wound healing and regenerative responses occurs via several factors including ECM proteins, growth factors, and cytokines, particularly of the TNF superfamily. In this review, we will discuss the role of two TNF superfamily members, lymphotoxin-β (LTβ), and TNF-like weak inducer of apoptosis (TWEAK), in regulating liver regeneration and wound healing.

## TWEAK/FN14 Signaling

### TWEAK/FN14 signaling induces LPC proliferation via NFκB activation

TWEAK ligand was first identified as a novel cell-associated and secreted factor with TNF family homology, which induced cytotoxicity in the human adenocarcinoma cell line HT29 in combination with IFNϒ treatment (28). TWEAK interacts with target cells via its receptor, fibroblast growth factor-inducible 14 (Fn14) (29), which is highly homologous in mouse and human tissues, and is upregulated in HCC lines and tissues (30). Biologically, TWEAK has been shown to regulate numerous cellular processes including proliferation, differentiation, migration, and cell survival and has also been described as a pro-angiogenic and pro-inflammatory factor (31). In chronic liver injury and repair, the principal function of TWEAK appears to initiate ductal proliferation and LPC expansion via activation of NFκB signaling. Ductal hyperplasia is observed in livers of mice overexpressing TWEAK under the control of the liver-specific α1-antitrypsin promoter, demonstrating the ability of TWEAK signaling, in isolation, to initiate ductal expansion (32). Conversely, ductal cell expansion is mitigated after pharmacological blocking of TWEAK signaling as well as in Fn14-knockout mice subjected to either 3,5-diethoxycarbonyl-1,4-dihydrocollidine (DDC) or choline-deficient, ethionine-supplemented (CDE)-induced chronic liver injury (22, 32). Furthermore, macrophages and natural killer cells have been shown to be the primary producers of TWEAK ligand in chronic liver injury, providing an important link between the inflammatory response and TWEAK-mediated ductal expansion (33). In the CDE model, TWEAK-producing macrophages have been observed in close association with expanding ductal cells (33), suggesting a mechanism whereby focal points of inflammatory cells signal via the TWEAK/Fn14 pathway to promote LPC expansion. Accordingly, transplantation of bone marrow-derived macrophages into normal liver stimulates a TWEAK-dependent response in LPCs and biliary cells, demonstrating a primary role of macrophage-generated TWEAK in initiating the DR (23). Previous studies indicate that this effect is mediated via TWEAK-induced activation of NFκB signaling (22).

The NFκB family comprises five monomers, which interact via N-terminal Rel homology domain (RHD) polypeptides. These monomers, RelA, RelB, cRel, p50, and p52, interact to form an active NFκB homo- or heterodimer and translocate to the nucleus to activate transcription of genes involved in numerous biological processes including, but not limited to, inflammation, proliferation, and cell survival (34). Regulation of nuclear localization occurs by sequestering of NFκB dimers through binding with inhibitory proteins (IκBs). Inhibition is released through phosphorylation and subsequent proteolytic degradation of IκB. Phosphorylation of IκB is initiated by binding of extracellular mediators to their receptors, which lead to activation of IκB-kinase (IKK) via NFκB essential modulator (NEMO)-dependent (canonical) or independent (non-canonical) mechanisms (35). NFκB plays a vital role in all aspects of chronic liver diseases (CLD), acting as a transducer of cytokine-mediated signals promoting inflammation and fibrosis (36), particularly in the survival and activation of HSCs (37, 38). NFκB signaling is activated upon TNF treatment of LPCs, promoting its mitogenic effects (39), and also regulates IL-6/TNF-mediated upregulation of LTβ in LPCs (40, 41).

TWEAK has been shown to activate NFκB via canonical (42) and non-canonical mechanisms (43). Accordingly, strong cytoplasmic and nuclear NFκB is observed in proliferating pan cytokeratin-expressing cholangiocytes and LPCs following recombinant TWEAK administration in CDE-injured mice (22). *In vitro* studies in the LPC lines BMOL and BMOL-TAT demonstrated dose-dependent proliferation following TWEAK stimulation. Mitogenesis was inhibited by transfection with siRNA targeting the NFκB p50 subunit, confirming the pro-proliferative effect of TWEAK in LPCs is NFκB-dependent (22). The ability of TWEAK to promote proliferation and self-renewal of LPCs appears to be vital in allowing LPCs to survive and thrive in the chronic liver injury setting. Interestingly, this modulation also affects the dynamics of hepatic fibrosis and inflammation.

### TWEAK as a regulator of fibrogenesis

Assessing the cellular players involved in TWEAK signaling provides unique insights into its versatile role in establishing a niche conducive to wound healing, regeneration as well as carcinogenesis. In addition to stimulating ductal/LPC expansion, one of the therapeutically interesting consequences of altering TWEAK signaling in chronic liver injury is the co-modulation of liver fibrosis. Experiments utilizing TWEAK pathway knockout mice and exogenous TWEAK stimulation/inhibition have shown a positive correlation between LPC proliferation and the fibrogenic response to chronic liver injury. Collagen deposition and the expression of TIMPs are reduced in Fn14-knockout mice on the CDE diet (22). Similarly, a recent study of TWEAK modulation in CCl_4_-stimulated fibrosis with partial hepatectomy (PHx)-induced hepatocyte deficit has shown that pharmacological TWEAK inhibition decreases collagen deposition during LPC expansion following PHx (44).

Given that the main function of TWEAK signaling appears to be in activating LPC proliferation, how might the TWEAK pathway regulate the fibrotic response of the liver? One possibility that is yet to be investigated is that TWEAK could act directly on fibrosis-driving HSCs. Supportive evidence for this possible scenario is provided by results showing that at least a subset of freshly isolated, activated HSCs express the receptor Fn14 and are therefore potentially TWEAK-responsive in CDE-induced liver injury (22). Another intriguing possibility is that modulating TWEAK/Fn14 signaling could influence the potency of the fibrogenic and inflammatory responses by modulating ductal/LPC cross-talk with leukocytes and/or HSCs. In the next section of this review, we will explore the mechanisms by which TWEAK signaling might regulate the inflammatory and fibrogenic responses through its effects on the LPC compartment.

### TWEAK regulation of hepatic inflammation

Through its effects on LPC numbers, TWEAK/Fn14 signaling could affect the dynamics of inflammation in chronic liver injury via LPC-mediated recruitment of inflammatory cells. Fn14-deficient mice fed a CDE diet display a delayed response of CD45^+^ (general inflammatory) and F4/80^+^ (macrophage) leukocytes. Conversely, stimulation of LPC proliferation and the DR with recombinant TWEAK injections results in an increase in CD45^+^ cells (22), suggesting that the amplitude of the DR might have an impact on the level of inflammatory cell recruitment. Given that LPCs express chemokine (C–C motif) ligand 2 (CCL2), also referred to as monocyte chemotactic protein-1 (MCP-1), and chemokine (C-X3-C motif) ligand 1 (CX_3_CL1), and have a demonstrated ability to attract CD11b^+^ macrophages isolated from normal and CDE-injured livers *in vitro* (33), the dynamics of the DR may play a role in regulating the inflammatory response. Since inflammatory cells, particularly the macrophage compartment, support the establishment of fibrosis in chronic liver injury by affecting HSC activation (45), influences on inflammatory cell recruitment may also affect the dynamics of liver fibrosis initiation and injury progression. Consequently, targeting the TWEAK/Fn14 pathway may be an effective way to alter the wound healing response by influencing the dynamics of LPC/inflammatory cell/HSC cross-talk.

## LPC/HSC Cross-Talk via LTβ Signaling

In an effort to understand the opposing roles of LPCs and HSCs in controlling regeneration and wound healing versus fibrogenesis progression and carcinogenesis, pathways involved in LPC/HSC cross-talk are currently under investigation. As we have discussed, modulating the LPC response via TWEAK signaling affects the dynamics of liver fibrosis mediated by HSCs. Additionally, Boulter *et al*. demonstrated regulation of LPC differentiation by activated HSCs through expression of the Notch ligand Jagged 1 and Notch-dependent biliary specification in adjacent LPCs (24). Conversely, expression of MCP-1 by cholangiocytes within hyperplastic or mature bile ducts was shown to drive HSC/myofibroblast chemotaxis in chronic cholestatic liver disease (25). These studies demonstrate the clear interactions of LPCs and HSCs during progressive chronic liver injury. Thus, interventions targeting pathways modulating LPC/HSC interactions might be of therapeutic benefit in patients with chronic liver disease. One such novel target is the TNF family member LTβ, which was recently discovered as a key regulator of LPC/HSC cross-talk, facilitated by NFκB-dependent downstream signaling (13).

LTβ is a type II transmembrane protein that signals as a cell surface-anchored heterotrimer with LTα (i.e., LTα2:β1 or predominantly LTα1:β2) (46). LTβ levels are increased in various animal chronic liver injury models including bile duct ligation (47) and CDE-induced injury (48, 49) and its expression correlates with the severity of fibrosis in chronic HCV infection in humans (50). It has been demonstrated on the surface of cells of the lymphocytic lineage, including activated B and T cells as well as natural killer cells (51) but interestingly also on small portal hepatocytes and proliferating LPCs during chronic liver injury (50). In our CDE injury model, these LTβ^+^ LPCs are observed in close proximity to activated HSCs, which express the LTβ receptor (LTβR). *In vitro* studies revealed that upon receptor binding, LTβ initiates a NFκB-dependent signaling cascade in HSCs that results in expression of chemotaxis-associated mediators ICAM-1 and RANTES, which in turn recruit RANTES receptor (C–C chemokine receptor type 5, CCR5)-positive LPCs (13). Hence this paracrine cytokine/chemokine cross-talk has the capacity to facilitate LPC and HSC migration through the liver parenchyma to sites of injury in addition to promoting wound healing by recruitment of new leukocytes, LPCs and HSCs. The LTβ signaling pathway also plays a role in fibrogenesis, since chronically injured LTβR knockout mice show reduced numbers of αSMA^+^ HSCs and decreased collagen deposition as evidenced by reduced Sirius Red staining (13). Concurrently, numbers of A6^+^ and muscle pyruvate kinase 2 (M_2_PK)^+^ LPCs are reduced (48), which once again suggests the co-regulation of the fibrogenic and the progenitor cell response in chronic liver injury. The significance of the LTβ pathway in liver disease and hepatocarcinogenesis was highlighted by Haybaeck et al. who analyzed tg1223 mice, which overexpress lymphotoxin in the liver, and showed that sustained expression leads to chronic hepatitis and eventually to HCC. To confirm results, LTβ overexpression was neutralized by pharmacological blocking of LTβR, which drastically reduced liver injury and prevented HCC formation (19). Hence, suppression of this pathway might be beneficial in liver diseases with a chronic overexpression of cytokines that signal through LTβR, including LTα and LTβ or LTβ-related inducible ligand competing for glycoprotein D binding to herpes virus entry mediator on T cells (LIGHT), such as seen in chronic HBV or HCV infection.

## Therapeutic Potential and Future Directions

In the Western world, chronic liver injury is ever increasing in prevalence. A variety of etiologies including chronic HBV/HCV infection, and non-alcoholic fatty liver disease can cause fibrosis and, subsequently, cirrhosis and HCC. The World Health Organization ranks CLD as the ninth commonest global cause of death with end organ failure as a result of cirrhosis and HCC, accounting for half the mortality each (52). Most HCC cases arise in the setting of established cirrhosis, with a median survival of 6–16 months, if untreated (53). At present, a range of primary treatment options including antiviral therapy and weight reduction strategies are variably effective in these conditions. Unfortunately, a significant number of patients still progress to end-stage liver disease and many require orthotopic liver transplantation. However, limited availability of donor organs and religious and/or economic reasons may restrict access to transplantation surgery. Prolonged waiting times for donor organs often result in disease progression and death of patients with initially treatable disease. Thus, the development of new therapeutic strategies for the prevention or treatment of hepatic fibrosis and its sequelae of cirrhosis and HCC are urgently required.

The carcinogenic and fibrogenic processes are amenable to manipulation by agents, which interfere with these processes, however to date approaches have been limited, and new targeted therapies such as tyrosine kinase inhibitors have not significantly improved survival (54, 55). Identification of new cellular targets for preventative therapies that minimize fibrosis or carcinogenesis represent the future for advancement of therapy, and knowledge of cross-talk and signaling pathways such as those defined here are critical for such advancement. The key will be balancing beneficial effects on reduced fibrosis and carcinogenesis against detrimental effects consequential to impaired replacement of healthy hepatocytes or cholangiocytes, as can be seen following IFN-based therapies for chronic viral hepatitis (56).

## Summary

The liver responds to chronic injury by initiating an inflammatory response, which enables the dual action of resolving injury, through the activation of fibrosis, and regeneration of injured tissue comprising the activation and differentiation of LPCs. These processes are inextricably linked and act in concert to reinforce and progress the chronic injury response until such a time that injury abates. Since these processes are linked, affecting the biology of one necessarily affects the dynamics of the whole system. As we have discussed in this review, TWEAK/Fn14 signaling plays a primary role in regulating LPC expansion and in doing so, affects both the inflammatory and wound healing responses of the liver through cellular cross-talk. Through interactions between LPCs and the immune system, TWEAK modulation of LPC numbers may act to enhance the inflammatory response at the site of regeneration, reinforcing LPC expansion. Likewise, LPC interactions with HSCs via LTβ signaling may also promote recruitment of leukocytes to the site of injury, amplifying the fibrogenic response of the liver. Accordingly, TWEAK signaling may be an important “valve” with which we might modulate the landscape of chronic liver injury. Therefore, we propose that therapies targeting TWEAK signaling in chronic liver injury may be useful for reducing the severity of fibrosis through its dual action on the inflammatory and HSC compartments via LPCs. In doing so, it may be possible to diminish progression to cirrhosis and liver failure, as well as limiting the pro-tumorigenic environment existing in the regenerative niche supporting LPCs.

## Conflict of Interest Statement

The authors declare that the research was conducted in the absence of any commercial or financial relationships that could be construed as a potential conflict of interest.

## References

[1] Theise ND Saxena R Portmann BC Thung SN Yee H Chiriboga L The canals of hering and hepatic stem cells in humans. Hepatology (1999) 30(6):1425–33 10.1002/hep.510300614 10573521

[2] Roskams TA Theise ND Balabaud C Bhagat G Bhathal PS Bioulac-Sage P Nomenclature of the finer branches of the biliary tree: canals, ductules, and ductular reactions in human livers. Hepatology (2004) 39(6):1739–45 10.1002/hep.20130 15185318

[3] Furuyama K Kawaguchi Y Akiyama H Horiguchi M Kodama S Kuhara T Continuous cell supply from a Sox9-expressing progenitor zone in adult liver, exocrine pancreas and intestine. Nat Genet (2011) 43(1):34–41 10.1038/ng.722 21113154

[4] Espanol-Suner R Carpentier R Van Hul N Legry V Achouri Y Cordi S Liver progenitor cells yield functional hepatocytes in response to chronic liver injury in mice. Gastroenterology (2012) 143(6):1564.e–75.e 10.1053/j.gastro.2012.08.024 22922013

[5] Viebahn CS Tirnitz-Parker JE Olynyk JK Yeoh GC Evaluation of the “Cellscreen” system for proliferation studies on liver progenitor cells. Eur J Cell Biol (2006) 85(12):1265–74 10.1016/j.ejcb.2006.08.006 17049406

[6] Knight B Tirnitz-Parker JE Olynyk JK C-kit inhibition by imatinib mesylate attenuates progenitor cell expansion and inhibits liver tumor formation in mice. Gastroenterology (2008) 135(3):969–79 10.1053/j.gastro.2008.05.077 18602920

[7] Brooling JT Campbell JS Mitchell C Yeoh GC Fausto N Differential regulation of rodent hepatocyte and oval cell proliferation by interferon gamma. Hepatology (2005) 41(4):906–15 10.1002/hep.20645 15799032

[8] Nguyen LN Furuya MH Wolfraim LA Nguyen AP Holdren MS Campbell JS Transforming growth factor-beta differentially regulates oval cell and hepatocyte proliferation. Hepatology (2007) 45(1):31–41 10.1002/hep.21466 17187411

[9] Jung Y Witek RP Syn WK Choi SS Omenetti A Premont R Signals from dying hepatocytes trigger growth of liver progenitors. Gut (2010) 59(5):655–65 10.1136/gut.2009.204354 20427400 PMC3632642

[10] Grzelak CA Martelotto LG Sigglekow ND Patkunanathan B Ajami K Calabro SR The intrahepatic signalling niche of hedgehog is defined by primary cilia positive cells during chronic liver injury. J Hepatol (2014) 60(1):143–51 10.1016/j.jhep.2013.08.012 23978713

[11] Mederacke I Hsu CC Troeger JS Huebener P Mu X Dapito DH Fate tracing reveals hepatic stellate cells as dominant contributors to liver fibrosis independent of its aetiology. Nat Commun (2013) 4:2823 10.1038/ncomms3823 24264436 PMC4059406

[12] Kessenbrock K Plaks V Werb Z Matrix metalloproteinases: regulators of the tumor microenvironment. Cell (2010) 141(1):52–67 10.1016/j.cell.2010.03.015 20371345 PMC2862057

[13] Ruddell RG Knight B Tirnitz-Parker JE Akhurst B Summerville L Subramaniam VN Lymphotoxin-beta receptor signaling regulates hepatic stellate cell function and wound healing in a murine model of chronic liver injury. Hepatology (2009) 49(1):227–39 10.1002/hep.22597 19111021

[14] Lowes KN Brennan BA Yeoh GC Olynyk JK Oval cell numbers in human chronic liver diseases are directly related to disease severity. Am J Pathol (1999) 154(2):537–41 10.1016/S0002-9440(10)65299-6 10027411 PMC1849988

[15] Sobaniec-Lotowska ME Lotowska JM Lebensztejn DM Ultrastructure of oval cells in children with chronic hepatitis B, with special emphasis on the stage of liver fibrosis: the first pediatric study. World J Gastroenterol (2007) 13(21):2918–22 17589940 10.3748/wjg.v13.i21.2918 PMC4171142

[16] Nobili V Carpino G Alisi A Franchitto A Alpini G De Vito R Hepatic progenitor cells activation, fibrosis, and adipokines production in pediatric nonalcoholic fatty liver disease. Hepatology (2012) 56(6):2142–53 10.1002/hep.25742 22467277

[17] Clouston AD Powell EE Walsh MJ Richardson MM Demetris AJ Jonsson JR Fibrosis correlates with a ductular reaction in hepatitis C: roles of impaired replication, progenitor cells and steatosis. Hepatology (2005) 41(4):809–18 10.1002/hep.20650 15793848

[18] Roskams T De Vos R Desmet V ‘Undifferentiated progenitor cells’ in focal nodular hyperplasia of the liver. Histopathology (1996) 28(4):291–9 10.1046/j.1365-2559.1996.d01-438.x 8732337

[19] Haybaeck J Zeller N Wolf MJ Weber A Wagner U Kurrer MO A lymphotoxin-driven pathway to hepatocellular carcinoma. Cancer Cell (2009) 16(4):295–308 10.1016/j.ccr.2009.08.021 19800575 PMC4422166

[20] Lee ES Han EM Kim YS Shin BK Kim CH Kim HK Occurrence of c-kit+ tumor cells in hepatitis B virus-associated hepatocellular carcinoma. Am J Clin Pathol (2005) 124(1):31–6 10.1309/LETTWN3LUF516HR0 15923163

[21] Lee JS Heo J Libbrecht L Chu IS Kaposi-Novak P Calvisi DF A novel prognostic subtype of human hepatocellular carcinoma derived from hepatic progenitor cells. Nat Med (2006) 12(4):410–6 10.1038/nm1377 16532004

[22] Tirnitz-Parker JE Viebahn CS Jakubowski A Klopcic BR Olynyk JK Yeoh GC Tumor necrosis factor-like weak inducer of apoptosis is a mitogen for liver progenitor cells. Hepatology (2010) 52(1):291–302 10.1002/hep.23663 20578156

[23] Bird TG Lu WY Boulter L Gordon-Keylock S Ridgway RA Williams MJ Bone marrow injection stimulates hepatic ductular reactions in the absence of injury via macrophage-mediated TWEAK signaling. Proc Natl Acad Sci U S A (2013) 110(16):6542–7 10.1073/pnas.1302168110 23576749 PMC3631632

[24] Boulter L Govaere O Bird TG Radulescu S Ramachandran P Pellicoro A Macrophage-derived Wnt opposes Notch signaling to specify hepatic progenitor cell fate in chronic liver disease. Nat Med (2012) 18(4):572–9 10.1038/nm.2667 22388089 PMC3364717

[25] Ramm GA Shepherd RW Hoskins AC Greco SA Ney AD Pereira TN Fibrogenesis in pediatric cholestatic liver disease: role of taurocholate and hepatocyte-derived monocyte chemotaxis protein-1 in hepatic stellate cell recruitment. Hepatology (2009) 49(2):533–44 10.1002/hep.22637 19115220

[26] Boulter L Lu WY Forbes SJ Differentiation of progenitors in the liver: a matter of local choice. J Clin Invest (2013) 123(5):1867–73 10.1172/JCI66026 23635784 PMC3635730

[27] Tirnitz-Parker JE Olynyk JK Ramm GA Role of TWEAK in co-regulating liver progenitor cell and fibrogenic responses. Hepatology (2013). 10.1002/hep.26701 24038142

[28] Chicheportiche Y Bourdon PR Xu H Hsu YM Scott H Hession C TWEAK, a new secreted ligand in the tumor necrosis factor family that weakly induces apoptosis. J Biol Chem (1997) 272(51):32401–10 10.1074/jbc.272.51.32401 9405449

[29] Wiley SR Cassiano L Lofton T Davis-Smith T Winkles JA Lindner V A novel TNF receptor family member binds TWEAK and is implicated in angiogenesis. Immunity (2001) 15(5):837–46 10.1016/S1074-7613(01)00232-1 11728344

[30] Feng SL Guo Y Factor VM Thorgeirsson SS Bell DW Testa JR The Fn14 immediate-early response gene is induced during liver regeneration and highly expressed in both human and murine hepatocellular carcinomas. Am J Pathol (2000) 156(4):1253–61 10.1016/S0002-9440(10)64996-6 10751351 PMC1876890

[31] Burkly LC Michaelson JS Hahm K Jakubowski A Zheng TS TWEAKing tissue remodeling by a multifunctional cytokine: role of TWEAK/Fn14 pathway in health and disease. Cytokine (2007) 40(1):1–16 10.1016/j.cyto.2007.09.007 17981048

[32] Jakubowski A Ambrose C Parr M Lincecum JM Wang MZ Zheng TS TWEAK induces liver progenitor cell proliferation. J Clin Invest (2005) 115(9):2330–40 10.1172/JCI23486 16110324 PMC1187931

[33] Viebahn CS Benseler V Holz LE Elsegood CL Vo M Bertolino P Invading macrophages play a major role in the liver progenitor cell response to chronic liver injury. J Hepatol (2010) 53(3):500–7 10.1016/j.jhep.2010.04.010 20561705

[34] Hayden MS Ghosh S Shared principles in NF-kappaB signaling. Cell (2008) 132(3):344–62 10.1016/j.cell.2008.01.020 18267068

[35] Shih VF Tsui R Caldwell A Hoffmann A A single NFkappaB system for both canonical and non-canonical signaling. Cell Res (2011) 21(1):86–102 10.1038/cr.2010.161 21102550 PMC3193412

[36] Elsharkawy AM Mann DA Nuclear factor-kappaB and the hepatic inflammation-fibrosis-cancer axis. Hepatology (2007) 46(2):590–7 10.1002/hep.21802 17661407

[37] Gao R Brigstock DR Activation of nuclear factor kappa B (NF-kappaB) by connective tissue growth factor (CCN2) is involved in sustaining the survival of primary rat hepatic stellate cells. Cell Commun Signal (2005) 3:14 10.1186/1478-811X-3-14 16303051 PMC1308830

[38] Hellerbrand C Jobin C Iimuro Y Licato L Sartor RB Brenner DA Inhibition of NFkappaB in activated rat hepatic stellate cells by proteasome inhibitors and an IkappaB super-repressor. Hepatology (1998) 27(5):1285–95 10.1002/hep.510270514 9581682

[39] Kirillova I Chaisson M Fausto N Tumor necrosis factor induces DNA replication in hepatic cells through nuclear factor kappaB activation. Cell Growth Differ (1999) 10(12):819–28 10616907

[40] Subrata LS Lowes KN Olynyk JK Yeoh GC Quail EA Abraham LJ Hepatic expression of the tumor necrosis factor family member lymphotoxin-beta is regulated by interleukin (IL)-6 and IL-1beta: transcriptional control mechanisms in oval cells and hepatoma cell lines. Liver Int (2005) 25(3):633–46 10.1111/j.1478-3231.2005.01080.x 15910501

[41] Subrata LS Voon DC Yeoh GC Ulgiati D Quail EA Abraham LJ TNF-inducible expression of lymphotoxin-beta in hepatic cells: an essential role for NF-kappaB and Ets1 transcription factors. Cytokine (2012) 60(2):498–504 10.1016/j.cyto.2012.05.029 22742857

[42] Saitoh T Nakayama M Nakano H Yagita H Yamamoto N Yamaoka S TWEAK induces NF-kappaB2 p100 processing and long lasting NF-kappaB activation. J Biol Chem (2003) 278(38):36005–12 10.1074/jbc.M304266200 12840022

[43] Brown SA Richards CM Hanscom HN Feng SL Winkles JA The Fn14 cytoplasmic tail binds tumour-necrosis-factor-receptor-associated factors 1, 2, 3 and 5 and mediates nuclear factor-kappaB activation. Biochem J (2003) 371(Pt 2):395–403 10.1042/BJ20021730 12529173 PMC1223299

[44] Kuramitsu K Sverdlov DY Liu SB Csizmadia E Burkly L Schuppan D Failure of fibrotic liver regeneration in mice is linked to a severe fibrogenic response driven by hepatic progenitor cell activation. Am J Pathol (2013) 183(1):182–94 10.1016/j.ajpath.2013.03.018 23680654 PMC3702745

[45] Duffield JS Forbes SJ Constandinou CM Clay S Partolina M Vuthoori S Selective depletion of macrophages reveals distinct, opposing roles during liver injury and repair. J Clin Invest (2005) 115(1):56–65 10.1172/JCI22675 15630444 PMC539199

[46] Ware CF VanArsdale TL Crowe PD Browning JL The ligands and receptors of the lymphotoxin system. Curr Top Microbiol Immunol (1995) 198:175–218 7774281 10.1007/978-3-642-79414-8_11

[47] Lee CM Knight B Yeoh GC Ramm GA Olynyk JK Lymphotoxin-beta production following bile duct ligation: possible role for Kupffer cells. J Gastroenterol Hepatol (2005) 20(11):1762–8 10.1111/j.1440-1746.2005.04065.x 16246198

[48] Akhurst B Matthews V Husk K Smyth MJ Abraham LJ Yeoh GC Differential lymphotoxin-beta and interferon gamma signaling during mouse liver regeneration induced by chronic and acute injury. Hepatology (2005) 41(2):327–35 10.1002/hep.20520 15660390

[49] Knight B Matthews VB Akhurst B Croager EJ Klinken E Abraham LJ Liver inflammation and cytokine production, but not acute phase protein synthesis, accompany the adult liver progenitor (oval) cell response to chronic liver injury. Immunol Cell Biol (2005) 83(4):364–74 10.1111/j.1440-1711.2005.01346.x 16033531

[50] Lowes KN Croager EJ Abraham LJ Olynyk JK Yeoh GC Upregulation of lymphotoxin beta expression in liver progenitor (oval) cells in chronic hepatitis C. Gut (2003) 52(9):1327–32 10.1136/gut.52.9.1327 12912866 PMC1773812

[51] Ware CF Crowe PD Grayson MH Androlewicz MJ Browning JL Expression of surface lymphotoxin and tumor necrosis factor on activated T, B, and natural killer cells. J Immunol (1992) 149(12):3881–8 1281193

[52] Mathers CD Loncar D Projections of global mortality and burden of disease from 2002 to 2030. PLoS Med (2006) 3(11):e442 10.1371/journal.pmed.0030442 17132052 PMC1664601

[53] Bruix J Sherman M Practice Guidelines Committee AAftSoLD. Management of hepatocellular carcinoma. Hepatology (2005) 42(5):1208–36 10.1002/hep.20933 16250051

[54] Llovet JM Ricci S Mazzaferro V Hilgard P Gane E Blanc JF Sorafenib in advanced hepatocellular carcinoma. N Engl J Med (2008) 359(4):378–90 10.1056/NEJMoa0708857 18650514

[55] Llovet JM Schwartz M Mazzaferro V Resection and liver transplantation for hepatocellular carcinoma. Semin Liver Dis (2005) 25(2):181–200 10.1055/s-2005-871198 15918147

[56] Lim R Knight B Patel K McHutchison JG Yeoh GC Olynyk JK Antiproliferative effects of interferon alpha on hepatic progenitor cells in vitro and in vivo. Hepatology (2006) 43(5):1074–83 10.1002/hep.21170 16628647

